# Correspondence

**Published:** 1895-04

**Authors:** F. M. Perry

**Affiliations:** Houlton, Maine


### CORRESPONDENCE.

---

Dear Mr. Editor: I notice in the February issue of the Journal an interesting paragraph among the editorials on the subject of "The Necessity for a Uniform Professional Degree or Title," in which the position is taken that the degree or appellation V.S., or the words Veterinary Surgeon, would be quite sufficient for the practitioner of our science. Would you allow me in all charitableness and kindness just a word in regard to the same? Professional degrees or titles have generally been rendered in Latin from time immemorial, and I can see no reason why we, as a branch of one of the learned professions, should break precedent in this regard. In the first place, there is no such thing as a veterinary surgeon any more than a veterinary dentist, etc., there being no schools or colleges for the special training of those two branches. We are all veterinary surgeons, veterinary physicians, and veterinary dentists, for that matter.

Granting that to be the case, the title Veterinary Surgeon would certainly be a misnomer, and consequently the misuse of the device V.S. becomes at once apparent. D.V.S. would come nearer the point, provided it was intended to stand for "Doctor of Veterinary Science" (not surgery). But even this would be a little far-fetched; and also to use the letters V.M.D., as given in this order, would simply be to put the cart before the horse, which, figuratively as well as literally, is always confusing.

Medicine is the word used to express medical science in general, and one who has graduated from a college in which a general course of medical science is given receives the degree of "Medicinæ Doctoris," the abbreviation of which to M.D. is so familiar to us all. Now, the veterinarian is a graduate from a similar course in (veterinary) medical science, and therefore what else can he receive but the degree of "Medicinæ Doctoris Veterinaire," which, abbreviated, would, of course, be read M.D.V., and not V.M.D., nor D.M.V. This is simply in accordance with the rules of grammar and the traditions of the (human) medical profession, which are far older than our established branch of the science, and which we as yet have no reason to change. If we do nothing else, let us start right by hanging out a shingle that, while simple and plain, shall be worded and lettered correctly, according to the regulations of etymology and syntax, for we can in no wise afford to sacrifice proper construction and clearness for mere simplicity. Let us no longer, then, insult the intelligence of the educated public by displaying mystifying and absurd titles, but instead present to them on our door-plates a clear-cut appellation that shall convey its exact and true meaning. F. M. PERRY, M.D.V.

HOULTON, MAINE.

